# Light-Activated Protoporphyrin IX-Based Polysilsesquioxane Nanoparticles Induce Ferroptosis in Melanoma Cells

**DOI:** 10.3390/nano11092324

**Published:** 2021-09-07

**Authors:** Hemapriyadarshini Vadarevu, Ridhima Juneja, Zachary Lyles, Juan L. Vivero-Escoto

**Affiliations:** 1 Department of Chemistry, The University of North Carolina at Charlotte, Charlotte, NC 28223, USA; hvadarev@uncc.edu (H.V.); ridhima.juneja@gmail.com (R.J.); Zklyles@gmail.com (Z.L.); 2 Nanoscale Science Program, The University of North Carolina at Charlotte, Charlotte, NC 28223, USA; 3 The Center for Biomedical Engineering and Science, The University of North Carolina at Charlotte, Charlotte, NC 28223, USA

**Keywords:** photodynamic therapy, cancer treatment, cell death mechanisms, melanoma, nanomedicine

## Abstract

The use of nanoparticle-based materials to improve the efficacy of photodynamic therapy (PDT) to treat cancer has been a burgeoning field of research in recent years. Polysilsesquioxane (PSilQ) nanoparticles with remarkable features, such as high loading of photosensitizers, biodegradability, surface tunability, and biocompatibility, have been used for the treatment of cancer in vitro and in vivo using PDT. The PSilQ platform typically shows an enhanced PDT performance following a cell death mechanism similar to the parent photosensitizer. Ferroptosis is a new cell death mechanism recently associated with PDT that has not been investigated using PSilQ nanoparticles. Herein, we synthesized a protoporphyrin IX (PpIX)-based PSilQ platform (PpIX-PSilQ NPs) to study the cell death pathways, with special focus on ferroptosis, during PDT in vitro. Our data obtained from different assays that analyzed Annexin V binding, glutathione peroxidase activity, and lipid peroxidation demonstrate that the cell death in PDT using PpIX-PSilQ NPs is regulated by apoptosis and ferroptosis. These results can provide alternative approaches in designing PDT strategies to enhance therapeutic response in conditions stymied by apoptosis resistance.

## 1. Introduction

Photodynamic therapy (PDT) is a minimally invasive treatment for cancer and other diseases. PDT uses non-toxic photosensitizers (PSs) that, upon activation with light of a specific wavelength in the presence of cellular oxygen, trigger a photochemical process leading to the generatio n of reactive oxygen species (ROS) [1]. The efficiency of ROS production in PDT is governed by effective activation of the PS internalized by cells of interest. The ROS generated in PDT can consume intracellular antioxidant substances like glutathione (GSH). Most PSs form unstable aggregates in aqueous media due to hydrophobicity and often require the use of organic solvents to aid their solubility. The application of PSs for PDT is heavily dependent on aqueous stability, and the past decade has seen many efforts directed towards developing stable formulations of such molecules in water [2]. The use of nanoparticles (NPs) for physical or chemical encapsulation of hydrophobic PSs has been widely explored as an alternative approach to overcome these issues. Nanoplatforms, such as liposomes, polymeric systems, inorganic materials, and silica-based nanoparticles, have demonstrated their effectiveness as PS carriers [3,4]. Our group pioneered the use of polysilsesquioxane (PSilQ) materials as an efficient platform for the delivery of protoporphyrin (PpIX) for in-vitro and in-vivo treatment of cancer. We demonstrated the advantage of using a redox-responsive PpIX-PSilQ platform to enhance the PDT effect in vitro [5,6,7]. This approach was used to develop a degradable PpIX-PSilQ platform for the effective PDT treatment of triple negative breast cancer (TNBC) in vivo [8]. Furthermore, we assessed the multi-modal capability of PpIX-PSilQ NPs by combining PDT, chemotherapy, and gene silencing in the same platform for the treatment of TNBC [9]. Herein, we report the study of the cell death mechanisms associated with PpIX-PSilQ nanoparticles.

**Scheme 1:**
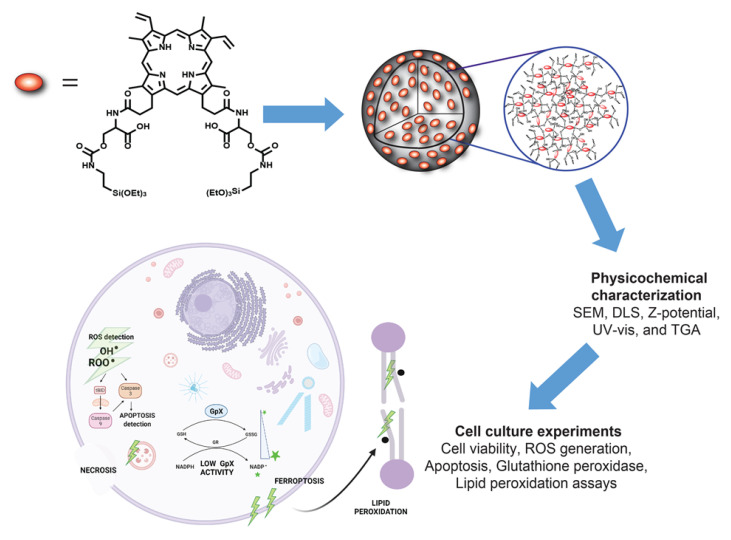
PpIX-PSilQ NPs are fabricated using the PpIX silane derivative (4) as a building block through microemulsion method. The physicochemical properties of the nanoparticles, such as SEM, DLS, ζ-potential, UV-vis, and TGA, are evaluated. The in-vitro performances, including the phototherapy, ROS generation, and internalization of PpIX-PSilQ NPs, are determined using A375 cells. The cell death mechanisms associated with the phototherapeutic outcome of PpIX-PSilQ NPs, like apoptosis, necrosis, and ferroptosis, against A375 cells are studied.

Apoptosis and necrosis are well characterized cell death mechanisms related to PDT [10]. Autophagy is a commonly reported cellular program in response to PDT that affords both cell survival and cell death depending on the cell type and degree of oxidative damage [11,12]. A newly discovered cell death mechanism is ferroptosis, which has shown to enhance the treatment efficacy of traditional chemo- and radiotherapy. Recently, ferroptosis has also been studied as an alternative mechanism of PDT to kill cancer cells [13]. We previously showed that apoptosis is the main mechanism linked to the phototoxicity of PpIX-PSilQ nanoparticles [8]; however, there is no study reporting the impact of ferroptosis in the PDT effect of this platform. Ferroptosis is an iron-dependent cell death that is caused by extreme peroxidation of cellular phospholipids and is a commonly reported response to tumor radiation therapy [14,15]. Excess hydroxyl radicals resulting from iron-catalyzed Fenton reactions can potentially initiate oxidative degradation of lipids in cell membranes by free-radical chain mechanism and subsequently cause cell death [15]. A recent study showed that GSH depletion associated to PDT could directly increase the accumulation of lipid peroxidation and enhance the ferroptosis effect [16,17]. Herein, we study the cell death mechanisms associated with PpIX-PSilQ nanoparticles, with special emphasis on ferroptosis (Scheme 1).

We synthesized PpIX-PSilQ nanoparticles for the purpose of this work. The physicochemical properties of this platform are similar to our previously reported systems [8,9]; however, this platform does not have stimuli-responsive features. Phototoxicity, generation of ROS, and cellular internalization were also confirmed using a malignant melanoma cell line, A375. Cell death mechanisms were investigated using different assays, including Annexin V apoptosis, glutathione peroxidase activity, and lipid peroxide probe C-11 BODIPY. The results show that PpIX-PSilQ nanoparticles in the presence of light produce cell death in A375 cells, which is correlated to apoptosis and ferroptosis. The role of ferroptosis in phototoxicity of the PpIX-PSilQ system was further confirmed using a ferroptosis inhibitor. These results demonstrated that ferroptosis is an important cell death mechanism associated to the PDT performance of PpIX-PSilQ nanoparticles.

## 2. Materials and Methods

### 2.1. Cell Culture

A375 cells were purchased from American Type Culture Collection (ATCC^®^ CRL-1619™, Manassas, VA, USA). The cells were cultured in DMEM with 10% FBS, 1% penicillin/streptomycin, 1% GlutaMAX, and 1% NEAAs at 37 °C with 5% CO_2_ atmosphere.

### 2.2. Stock Solutions for In-Vitro Experiments

For the treatment of cells with PpIX, 5 mM primary stock solution was freshly prepared in complete DMEM with 1% *v*/*v* of DMSO. A primary stock of Hoechst 33,342 at a concentration of 4 mM was prepared in deionized water containing 1% *v*/*v* DMSO and stored at −20 °C, which was diluted to 20 µM in complete DMEM for staining cells. Then, 10 mM DCFH-DA primary stock was prepared in DMSO and was diluted to 10 µM in complete DMEM before addition to cells. Then, 20 mM stock solution of Ferrostatin-1 was prepared in DMSO and was diluted to 2 µM in complete DMEM for cell treatment. A primary stock of PpIX-PSilQ NPs in a concentration equivalent to 200 µM of PpIX was prepared in complete DMEM by ultrasonication for 5 min and diluted for cell treatment.

### 2.3. Synthesis of PpIX-PSilQ Nanoparticles

The direct microemulsion method was used to synthesize the PpIX-PSilQ NPs [9]. The following procedure was used: 0.22 g of AOT (0.495 mmol) was added to 10 mL of nanopure water under gentle stirring for 30 min at room temperature. Then, 0.4 mL of n-butanol was added. Once the solution became clear, 0.1 mL of cyclohexane (oil phase) was added. The final mixture was stirred for 15 min to give a single-phase transparent solution. To this mixture, a solution of PpIX silane precursor (4, see Scheme S1) (1.6 mg) in 0.1 mL of 1:2 dimethyl sulfoxide (DMSO)/dimethyl formamide (DMF) mixture (*v*/*v*) was added dropwise under continuous stirring at room temperature. To enhance the solubility and condensation process of compound 4, 0.1 mL of aqueous ammonia (28%) followed by 0.2 mL of an aqueous solution of NaOH (2 M) was added to the solution. The reaction mixture was stirred for 48 h at room temperature. The nanoparticles were obtained by disrupting the microemulsion with an excess of acetone, which afforded the precipitation of the PpIX-PSilQ NPs. The final nanoparticles were collected by centrifugation (13,000 rpm for 10 min), followed by sequential washing steps with acetone, ethanol, and DMF to remove any unreacted reagents. The washed PpIX-PSilQ NPs were stored in ethanol at 4 °C.

### 2.4. Determination of Intracellular Reactive Oxygen Species (ROS)

A375 cells were seeded in six-well plates at a density of 5 × 10^4^ cells/well in 2 mL of complete DMEM and incubated at 37 °C in 5% CO_2_ atmosphere. After 24 h, PpIX-PSilQ nanoparticles (50 or 100 µM equivalent amount of PpIX) or free PpIX (10 or 50 µM) were added to the wells in fresh media. Followed by 48 h of incubation, the media was removed, and cells were washed with Dulbecco phosphate buffer solution (DPBS). Cell permeable ROS probe (DCFH-DA, 10 µM) was added to the cells in serum free media, and cells were incubated for 30 min at 37 °C and 5% CO_2_. After removing the media, the cells were washed with DPBS and irradiated (630 nm, 24.5 mW/cm^2^) for 20 min. Subsequently, cells were harvested using trypsin and analyzed for green fluorescence associated with the oxidation product, 2’,7’-dichlorofluorescein (DCF), using a BD LSRFortessa flow cytometer. Untreated cells were also incubated with the ROS probe; the data were utilized as the negative control.

### 2.5. Evaluation of Intracellular ROS Using Confocal Microscopy

A375 cells were seeded in a six-well plate containing glass coverslips at a density of 5 × 10^4^ cells/well in 2 mL of complete DMEM and incubated at 37 °C in 5% CO_2_ atmosphere for 24 h. After media removal, 50 µg/mL of PpIX-PSilQ NPs (equivalent to 25 µM PpIX) were added to the wells in fresh media and incubated for 48 h. Cells were washed once with DPBS, followed by the addition of 10 µM of ROS probe DCFDA in serum free media, and incubated for 30 min. Next, cells were washed once with DPBS and irradiated (630 nm, 24.5 mW/cm^2^) for 20 min. Coverslips were then mounted on to glass slides using adhesive spacers after adding 30 µL of DPBS to the slide. Images were acquired using Olympus FluoView 1000 confocal fluorescence microscope. A375 cells inoculated with PpIX-PSilQ nanoparticles (not irradiated), and untreated cells in the presence of the ROS probe were imaged as controls.

### 2.6. Cellular Uptake of PpIX-PSilQ Nanoparticles

To evaluate the cellular uptake of PpIX-PSilQ NPs in A375 cells, the cells were seeded in 24-well plates at a density of 2 × 10^4^ cells/well in 500 µL of complete DMEM and incubated for 24 h at 37 °C in 5% CO_2_ atmosphere. PpIX-PSilQ NPs or PpIX was added to the wells in fresh media at concentrations corresponding to 25 or 50 µM of PpIX. After 24 h of incubation, media was removed, cells were washed twice with DPBS, and cells were harvested with trypsin. Collected cells were resuspended in 200 µL of DPBS and analyzed for red channel fluorescence using a BD LSR Fortessa flow cytometer. Untreated cells were employed as negative controls.

To further evaluate the cellular uptake of PpIX-PSilQ NPs in A375 cells using CLSM, the cells were seeded in a six-well plate with glass slides at a density of 5 × 10^4^ cells/well in complete DMEM. After 24 h of incubation at 37 °C in 5% CO_2_ atmosphere, the media was removed, and 50 µg/mL of PpIX-PSilQ NPs were added to the wells in fresh media. The cells were incubated for another 24 h, the cell media was removed, and cells were washed twice with DPBS. Cells were stained with 20 µM of Hoescht 33342 nuclear staining solution and incubated for 20 min at 37 °C. After removal of the dye-containing media, coverslips were washed with DPBS and then mounted on to glass slides using adhesive spacers. Images were acquired using Olympus FluoView 1000 confocal fluorescence microscope at 40× magnification.

### 2.7. In Vitro Evaluation of PDT Triggered Apoptosis

A375 cells were seeded in six-well plates at a density of 5 × 10^4^ cells/well in complete DMEM. Followed by 24 h of incubation at 37 °C in 5% CO_2_ atmosphere, PpIX-PSilQ NPs (equivalent concentrations of PpIX of 50 or 100 µM) or PpIX (10 or 50 µM) were added to the wells in fresh media. After 48 h of incubation, cell media was removed, and the cells were washed once with DPBS prior to irradiation with red light (630 nm, 24.5 mW/cm^2^) for 20 min. Fresh media was replenished, and cells were incubated for additional 24 h at 37 °C in 5% CO_2_ atmosphere. Treated cells were harvested with trypsin and tested using Annexin V staining (FITC Annexin V Apoptosis Detection Kit, BD Pharmingen^TM^, San Jose, CA, USA) as per manufacturer’s protocol to determine apoptotic cell population. Annexin V antibody was added to the cells dispersed in 0.1 X binding buffer, followed by incubation for 20 min at room temperature. Cells were pelletized by centrifugation (2500 rpm, 5 min), and unbound Annexin V was removed. After excess antibody removal, 0.1 X binding buffer was used to wash cells once and for redispersion. Cells were co-stained with a vital dye (SYTOX™ Blue dead cell stain, Invitrogen™, (Waltham, MA, USA)) and classified into live, FITC+/dead, and FITC+/live populations using a BD LSR Fortessa flow cytometer. Untreated cells and non-irradiated samples were employed as negative controls.

### 2.8. Inhibition of Ferroptosis

The phototoxicity of PpIX-PSilQ NPs was evaluated in the presence of a ferroptosis inhibitor (Ferrostatin-1) using MTS assay. A375 cells were seeded in 96-well plates at a density of 2 × 10^3^ cells/well in 100 µL of complete DMEM and incubated at 37 °C in 5% CO_2_ atmosphere for 24 h. After cell media removal, PpIX (10–200 µM) or PpIX-PSilQ NPs (equivalent concentrations of PpIX of 10–250 µM) were added in fresh media to the cells. Followed by 48 h of incubation, cells were washed once with 100 µL of DPBS and irradiated with red light (630 nm, 24.5 mW/cm^2^) for 20 min. Treated cells were replenished with 100 µL of fresh media containing 2 µM Fer-1 (0.01% *v*/*v* DMSO) and incubated for additional 24 h at 37 °C in 5% CO_2_ atmosphere. Control dark experiments were conducted in parallel with PpIX-PSilQ NPs or PpIX at the same concentrations but were maintained in the dark for the entire duration of the experiment. To determine the phototoxicity of the PDT treatment, the cells were washed once with DPBS, and 100 µL media was added along with 20 µL of CellTiter 96 solution to each well. The cells were incubated for 2 h at 37 °C in 5% CO_2_ atmosphere. Cell viability (%) was analyzed and calculated as described above. The IC_50_ values are determined using GraphPad Prism (v8.3.0 for Windows, La Jolla, CA, USA), fitting the viability data to a nonlinear regression.

### 2.9. Evaluation of NADPH/NADP^+^ Kinetics

A375 cells were seeded at a density of 5 × 10^4^ cells/well in complete DMEM in six-well plates. After 24 h of incubation at 37 °C in 5% CO_2_ atmosphere, PpIX-PSilQ NPs, or PpIX (10, 30, or 50 µM of PpIX) were added to the cells in fresh media. Followed by 48 h of incubation, cell media was removed, and cells were washed once with DPBS and irradiated with red light (630 nm, 24.5 mW/cm^2^) for 20 min. Fresh media was replenished, and cells were incubated for additional 2 h. Then, cells were washed once with DPBS and harvested. Collected cells were centrifuged for 5 min at 4 °C at 13,000× *g*. Supernatants were collected by transferring to new tubes. Non-irradiated samples and untreated cells were employed as controls. Samples were prepared as per assay protocol (Glutathione Peroxidase Activity Assay Kit, Fluorometric, Abcam ab21992) in a 96-well solid black plate in triplicate per sample. Fluorescence intensity (Ex/Em = 420/480) was monitored using a TECAN SPARK^®^ multimode microplate reader in kinetic mode for 60 min. Reaction rates were determined using GraphPad Prism (v8.3.0 for Windows, La Jolla, CA, USA), fitting the kinetic data to a non-linear curve (second order polynomial).

### 2.10. Measurement of Intracellular Lipid Peroxides

A375 cells were seeded in six-well plates at a density of 5 × 10^4^ cells/well in 2 mL of complete DMEM and incubated at 37 °C in 5% CO_2_ atmosphere. After 24 h, PpIX-PSilQ NPs or PpIX (50 µM of PpIX) were added to the cells in fresh media. Followed by 48 h of incubation, cell media was removed, and cells were washed once with DPBS. Cell-permeable lipid peroxide probe C-11 BODIPY (10 µM) was added to the cells in serum free media, and cells were incubated for 30 min at 37 °C and 5% CO_2_. After removing the media, the cells were washed once with DPBS and irradiated (630 nm, 24.5 mW/cm^2^) for 20 min. Cells were incubated for 6 h post irradiation and harvested using trypsin. The fluorescence was analyzed using a BD LSRFortessa flow cytometer. A shift from ~590 nm to ~510 nm was expected from the oxidation of the polyunsaturated butadienyl portion of the dye. Control experiments of cells untreated, PpIX-PSilQ NPs, and PpIX in the absence of light were also carried out.

The extent of lipid peroxidation was further confirmed using confocal microscopy. A375 cells were seeded in six-well plates at a density of 5 × 10^4^ cells/well in complete DMEM at 37 °C in 5% CO_2_ atmosphere. After 24 h of incubation, cell media was removed, and 50 µg/mL of PpIX-PSilQ NPs were added to the wells in fresh media. Cell were incubated for additional 24 h, cell media was removed, and cells were washed twice with DPBS. Cell-permeable lipid peroxide probe C-11 BODIPY (10 µM) was added to the cells in serum free media and incubated for 30 min at 37 °C and 5% CO_2_. After removal of the cell media, the cells were washed once with DPBS and irradiated (630 nm, 24.5 mW/cm^2^) for 20 min. Then, 6 h after irradiation, coverslips were washed with DPBS and then mounted on to glass slides using adhesive spacers. Untreated cells were used as the baseline, while cells treated with PpIX-PSilQ NPs and PpIX in the absence of light were imaged as control experiments. Images were acquired using Olympus FluoView 1000 confocal fluorescence microscope at 40× magnification.

### 2.11. Statistics

All experimental results in this study are reported as mean ± standard deviation (SD) unless mentioned otherwise. The hydrodynamic size and ζ-potential were carried out in triplicate. The amount of PpIX loaded to the PSilQ nanoparticles was analyzed in triplicate using different batches. Cellular uptake, Annexin V apoptosis/necrosis, ROS detection by DCFH-DA, and C-11 BODIPY oxidation using flow cytometry were measured with a minimum of 10,000 gated cells and quantified in triplicates. The statistical analysis for all experiments was performed with two-way ANOVA using Tukey’s multiple comparison test. All the statistical analysis was performed using GraphPad prism (v8.2.0) with a *p*-value < 0.05 considered to be statistically significant. For the cell viability studies, GraphPad Prism was used to calculate the IC_50_ values (*n* = 6).

## 3. Results

### 3.1. Synthesis and Characterization of PpIX-PSilQ NPs

**Figure 1:**
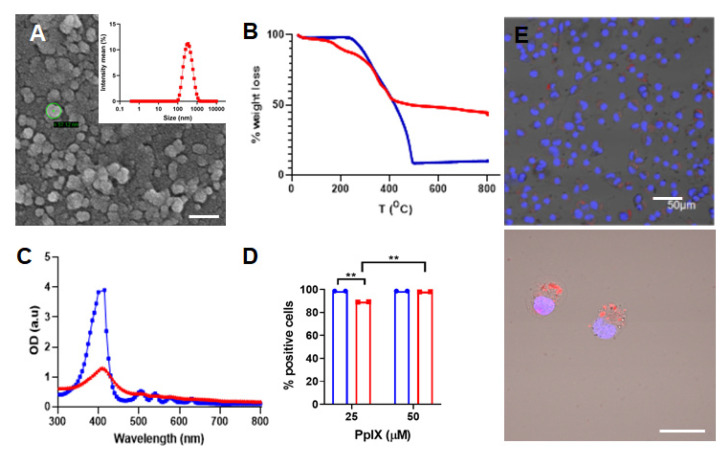
Characterization of PpIX-PSilQ NPs. (**A**) DLS and SEM image of PpIX-PSilQ NPs. Scale bar = 100 nm. (**B**) TGA plot for PpIX (blue) and PpIX-PSilQ NPs (red). (**C**) UV-vis spectrum of PpIX (blue) and PpIX-PSilQ NPs (red). (**D**) Flow cytometry data of PpIX-PSilQ nanoparticles (red) and free PpIX (blue) at different concentrations (25 and 50 µM PpIX) in A375 cells after 24 h of incubation. Statistics: two-way ANOVA using Tukey’s multiple comparison test; ** *p* ≤ 0.01. (**E**) Confocal microscopy images of A375 cells after 24 h incubation with PpIX-PSilQ NPs (25 µM PpIX). Image(s) show merged fluorescent channels of Hoechst-33342 nuclear stain dye (blue), PpIX fluorescence (red), and brightfield. Scale bars = 50 µm and 20 µm.

The synthesis of the PpIX silane precursor was carried out through a multi-step reaction pathway already reported by our group with slight modifications (Scheme S1) [8,9]. Compounds 2–4 were characterized using spectroscopic techniques as depicted in the Supporting Information. The structural properties of the PpIX-PSilQ nanoparticles were characterized using DLS, ζ-potential, and SEM. The hydrodynamic diameter of nanoparticles was determined as 262.6 ± 20.0 nm in PBS (Figure 1A). The ζ-potential measurements show a negative surface charge (−35.5 ± 4.0 mV) on the surface of PpIX-PSilQ NPs (Table S1). SEM images of PpIX-PSilQ NPs depict the nanoparticles as spherical in morphology with a size of 41.7 ± 4.9 nm (*n* = 10) (Figure 1A and Figure S1A). The loading capacity of PpIX in the nanoparticles was characterized using TGA, and the amount of PpIX was determined as 24.0 ± 2.0% wt (Figure 1B and Table S1), which was confirmed by UV-vis spectroscopy, 20.2 ± 3.6% wt (Figure S2). The UV-vis absorption spectrum corroborates the presence of PpIX in PpIX-PSilQ NPs (Figure 1C). The characteristic S-band for porphyrins was clearly observed at 404 nm. The colloidal and chemical stability of the nanoparticles was characterized using DLS and UV-vis spectroscopy, respectively. DLS data show that the hydrodynamic diameter and polydispersity index (PdI) of the PpIX-PSilQ NPs in complete cell media were fairly stable during 24 h (Figure S1B). The leak of PpIX molecules from PpIX-PSilQ NPs was studied in the presence and absence of a reducing agent (dithiothreitol = DTT) for 192 h. Minimal leakage (<10%) was determined during that time (Figure S1C).

### 3.2. In Vitro PDT Performance of PpIX-PSilQ NPs

The cellular internalization of PpIX-PSilQ nanoparticles was evaluated by flow cytometry and confocal microscopy using A375 cells. Flow cytometry data (Figure 1D) for PpIX-PSilQ NPs at two different concentrations of PpIX, 25 and 50 µM, showed 89.2 ± 0.1% and 98.5 ± 0.2% of positive cells, respectively. In the case of free PpIX, flow cytometry results showed over 99.0% of positive cells at the tested doses of 25 µM and 50 µM. Confocal micrographs obtained for PpIX-PSilQ nanoparticles at 25 µM confirmed the presence of red fluorescence spots inside A375 cells (Figure 1E).

The phototoxicity of PpIX-PSilQ NPs was evaluated in A375 cells in the presence of red light (630 nm; 30 J/cm^2^) for 20 min using the MTS assay. PpIX-PSilQ NPs showed dose-dependent phototoxicity, as seen in Figure S3A. The calculated IC_50_ value for nanoparticles was 81.2 µM. The cytotoxicity in the absence of light (dark cytotoxicity) of the nanoparticles was also tested. As seen in Figure S3B, PpIX-PSilQ NPs showed no cytotoxicity even at equivalent concentrations of PpIX as high as 250 µM. The phototoxicity of PpIX also depicted a dose-dependent response similar to PpIX-PSilQ NPs. The calculated IC_50_ for PpIX in the presence of red light was 9.4 µM. Dark cytotoxicity evaluation of free PpIX showed a 20–25% decline in cell viability in the concentration range of 100–250 µM.

**Figure 2:**
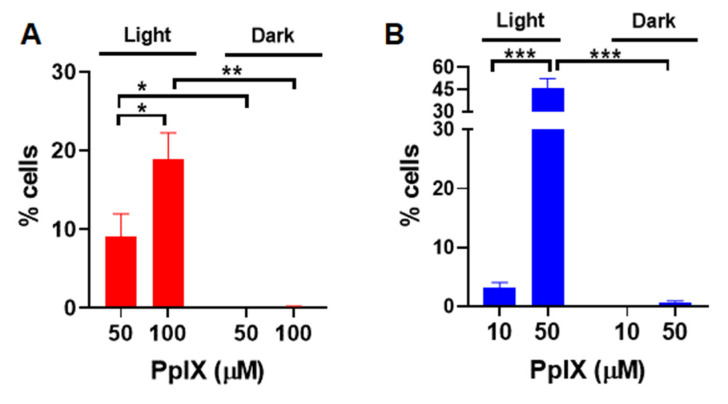
Reactive oxygen species generated by PpIX-PSilQ NPs. Quantification of ROS positive cells by flow cytometry of (**A**) PpIX-PSilQ nanoparticles (50 and 100 µM equivalent of PpIX) and (**B**) PpIX (10 and 50 µM) in the presence or absence of light. Data are represented as mean ± SD. Statistics: two-way ANOVA using Tukey’s multiple comparison test: *** *p* ≤ 0.001, ** *p* ≤ 0.01, * *p* ≤ 0.05, and ns: *p* > 0.05.

The production of some of ROS, including hydrogen peroxide, hydroxyl, and peroxyl radicals associated with PDT, were measured in vitro using a fluorescent ROS probe, DCFH-DA [18,19]. Upon diffusion into cells, DCFH-DA is deacetylated by cellular esterases to a non-fluorescent compound, which is later oxidized by ROS into fluorescent 2′,7′- dichlorofluorescein (DCF). A375 cells were treated with PpIX-PSilQ nanoparticles at 50 and 100 µM equivalent of PpIX. Quantification by flow cytometry shows 9.0 ± 2.9% and 18.9 ± 3.3% of DCF positive cells post irradiation for those concentrations, respectively (Figure 2A). In the case of PpIX, 3.2 ± 0.9% and 45.9 ± 6.6% of DCF positive cells were measured post irradiation for 10 and 50 µM, respectively (Figure 2B). For both PpIX-PSilQ NPs and PpIX, negligible production of ROS was detected in the absence of light irradiation. Confocal microscopy was used to visually confirm the generation of ROS. As seen in the confocal micrographs (Figure S3A,B), both PpIX-PSilQ nanoparticles and PpIX produce ROS inside the cells upon irradiation.

### 3.3. Apoptosis and Necrosis Induced by PpIX-PSilQ Nanoparticles

**Figure 3:**
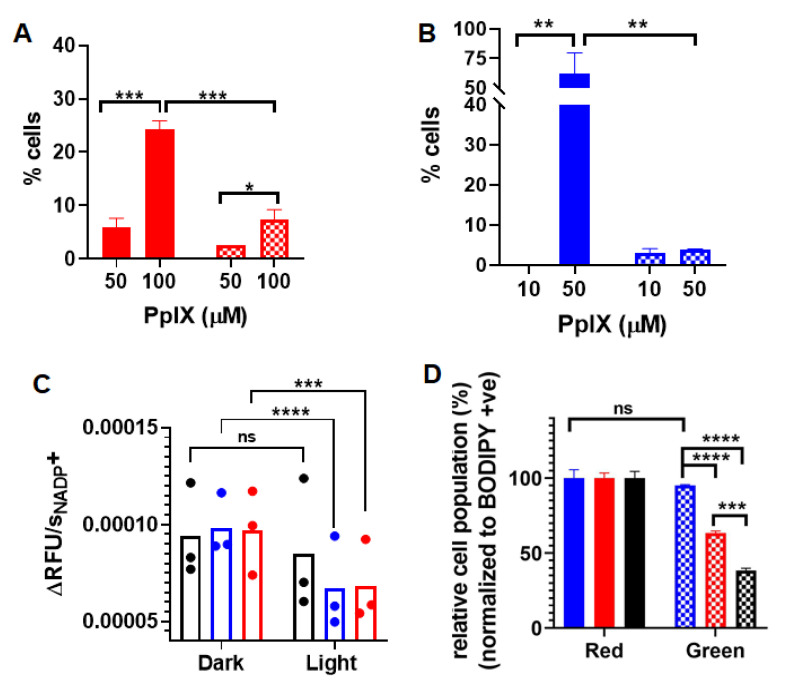
(**A**) Apoptotic (red solid bars) and necrotic (red checkered bars) cells after PDT treatment with PpIX-PSilQ NPs (50 and 100 µM equivalent of PpIX). (**B**) Apoptotic (blue solid bars) and necrotic (blue checkered bars) cells after PDT treatment with PpIX (10 and 50 µM). (**C**) Reaction rate values of NADP^+^ generation for control (black), PpIX-PSilQ NPs (50 µM PpIX eq.) (red), and PpIX (50 µM) (blue). (**D**) Quantification of lipid peroxidation using confocal microscopy in A375 cells treated with control (black), PpIX-PSilQ NPs (50 µM PpIX eq.) (red), and PpIX (50 µM) (blue) in the red (solid bars) and green (dashed bars) channels. Data are represented as mean ± SD. Statistics: two-way ANOVA using Tukey’s multiple comparison test: **** *p* ≤ 0.0001, *** *p* ≤ 0.001, ** *p* ≤ 0.01, * *p* ≤ 0.05, and ns: *p* > 0.05.

The generation of apoptosis and/or necrosis promoted by PDT using PpIX-PSilQ NPs was analyzed using flow cytometry in the presence of the SYTOX Blue dead-cell nuclear stain assay and Annexin V Apoptosis Detection Kit. SYTOX Blue dye penetrates compromised plasma membrane, staining nucleic acids inside the cells. The Annexin V assay contains a FITC-labeled antibody that binds to phosphatidylserine residues expressed on the plasma membrane of apoptotic cells. Two different concentrations of nanoparticles were evaluated in this experiment: 50 and 100 µM, based on the amount of PpIX. These concentrations were selected with the purpose of the photoactivity associated to the nanoparticles, which triggers a measurable response for the cell death mechanisms. Cells treated with nanoparticles in the presence of light showed 5.8 ± 1.7% and 24.2 ± 1.7% of Annexin-V-positive cells for 50 and 100 µM, respectively (Figure 3A). Dark controls for nanoparticles were used as negative controls, showing only 0.7 ± 0.6% and 4.7 ± 1.2% of Annexin-V-positive cells for the same concentrations (Figure S5A). A similar analysis was performed for PpIX at concentrations of 10 and 50 µM. As depicted in Figure 3B, cells treated with 50 µM of PpIX after light irradiation exhibited 62.3 ± 17.6% of Annexin-V-positive cells. PpIX at 10 µM did not show a significant production of apoptotic cells. Dark controls for PpIX show 3.7 ± 1.2% of Annexin-V-positive cells for 50 µM (Figure S5B). Cells treated with nanoparticles showed 2.6 ± 0.0% and 7.4 ± 1.8% necrotic cells for treatment concentrations 50 and 100 µM in the presence of light (Figure 3A). Nanoparticle treatment led to less than 0.1% necrotic cell death for 50 and 100 µM in the absence of light (Figure S5A). Necrosis was observed in 3.5 ± 2.3% and 4.9 ± 0.4% of the analyzed population of cells treated with 10 and 50 µM of free PpIX (Figure 3B) in the presence of light. In the case of dark controls, free PpIX treatment caused necrosis in 3.1 ± 1.0% and 3.9 ± 0.2% of cells treated with 10 and 50 µM, respectively (Figure S5B).

### 3.4. Inactivation of Glutathione Peroxidase Triggered by PpIX-PSilQ Nanoparticles

We assessed the effect of PpIX-PSilQ nanoparticles using A375 cells on the activity of the enzyme family glutathione peroxidase (GpX) indirectly through the NADPH oxidation reaction. The assay involves the oxidation of external glutathione (GSH) to glutathione disulfide (GSSG) catalyzed by GpX in collected cell lysates. The GSSG generated in the previous step is reduced to GSH by externally supplied glutathione reductase (GR) and nicotinamide adenine dinucleotide phosphate (NADPH), affording NADP^+^ as by-product (Figure S6A). The kinetics of formation of NADP^+^, monitored by the fluorescence of a NADP^+^-specific probe, is an indirect approach to measure the catalytic activity of GpX [20]. The kinetic profiles of NADP^+^ for cells incubated with PpIX-PSilQ nanoparticles or PpIX in the presence of light showed a clear reduction in the formation of NADP^+^ compared with control groups (Figure S6B). To confirm these results, the rates of formation of NADP^+^ were calculated (Figure 3C). The reaction rate values of cells treated with PpIX-PSilQ nanoparticles or PpIX indicate a significant decline in the generation of NADP^+^, which is as an indirect consequence in the reduction of the GpX activity.

### 3.5. Lipid ROS Generation Detected by a Lipid Peroxidation Sensor

**Figure 4:**
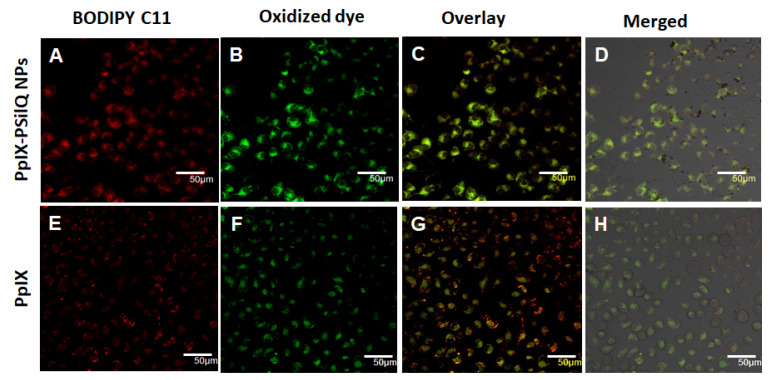
Lipid peroxidation detected by BODIPY™ 581/591 C-11 sensor. Confocal micrographs of A375 cells inoculated with (**A**–**D**) PpIX-PSilQ nanoparticles or (**E**–**H**) PpIX after light irradiation. The red channel depicts the presence of BODIPY 581/591 C-11 (**A**,**E**). The green channel shows the fluorescence corresponding to the oxidized version of BODIPY 581/591 C-11 (**B**,**F**). Merged image of the red and green channels (**C**,**G**) with the brightfield image (**D**,**H**). Scale bar = 50 µm.

The beginning of lipid peroxidation in cells is routinely characterized by measuring the oxidation of a lipophilic fluorescent probe, BODIPY™ 581/591 C11. The probe monitors the formation of oxygen-centered lipid radicals in phospholipid membranes of cells by eliciting an oxidation-induced shift in fluorescence emission peak from 590 nm (red) to 510 nm (green) [21]. We determined the impact on lipid peroxidation after treatment of A375 cells with PpIX-PSilQ nanoparticles by flow cytometry and confocal microscopy. The flow cytometry data in Figure 3D shows 24.9 ± 1.5% and 56.8 ± 1.2% increase in positive cells for PpIX-PSilQ nanoparticles and PpIX after irradiation as compared with control (two-way ANOVA, *p* ≤ 0.001). Control experiments with PpIX-PSilQ nanoparticles and PpIX in the absence of light showed less than 1% of positive cells (Figure S7). The lipid peroxidation in A375 cells associated with the PpIX-PSilQ nanoparticles after PDT treatment was further corroborated by confocal microscopy. Figure 4 shows the confocal micrographs associated with the BODIPY C11 dye (red channel), the oxidized version of the dye (green channel), and merged versions together with the brightfield. The red channel shows the presence of the BODIPY C11 dye in the membranes (Figure 4A,E). In the case of the samples that were irradiated, an enhancement in green fluorescence is observed for both PpIX-PSilQ NPs and PpIX (Figure 4B,F) as compared with control experiments in the absence of light (Figure S8). Merged images (Figure 4C,D,G,H) confirmed the overlap of the BODIPY C11 dye and its oxidized version after light irradiation.

### 3.6. Inhibition of Ferroptosis Using Ferrostatin-1

**Figure 5:**
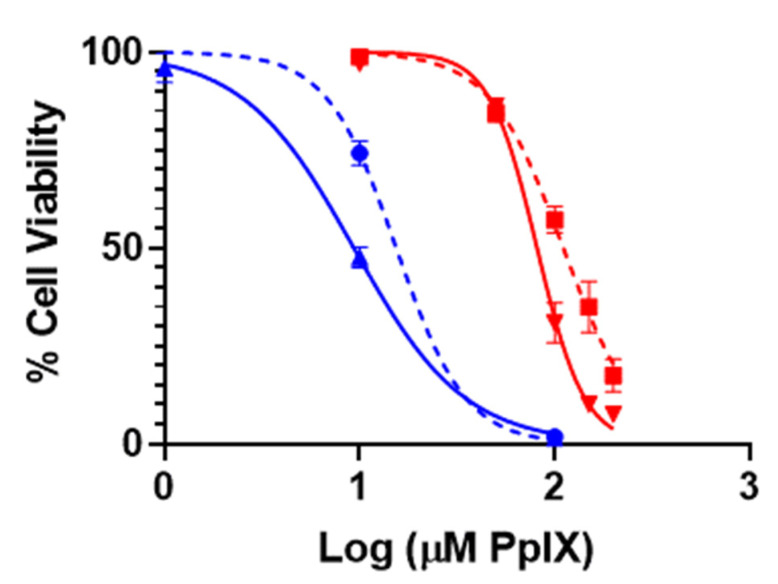
Phototoxicity of PpIX-PSilQ nanoparticles. PpIX-PSilQ nanoparticles (red) and PpIX (blue) treated A375 cells in the absence (solid) or presence (dashed) of Ferrostatin-1 (2 µM). Irradiation with red light (630 nm; 24.5 mW cm^−2^) for 20 min (*n* = 6).

To determine the impact of ferroptosis on the PDT treatment of A375 cells using PpIX-PSilQ nanoparticles, we evaluated the phototoxicity of the nanoparticles in the presence of a lipophilic antioxidant (Ferrostatin-1) (Figure 5). Ferrostatin-1 decreased the phototoxic effect of PpIX-PSilQ NPs on A375 cells by 36% as indicated by increase in the IC_50_ to 110.9 µM from 81.2 µM in the presence and absence of Ferrostatin-1, respectively. In the case of PpIX, the influence of Ferrostatin-1 shows an important reduction of 63% on the PDT effect against A375 cells with IC_50_ values of 15.4 µM and 9.4 µM in the presence and absence of Ferrostatin-1, respectively. Control experiments in the absence of light showed no cytotoxic effect associated with the presence of Ferrostatin-1 (Figure S8).

## 4. Discussion

Photodynamic therapy triggers different type of cell death mechanisms, with the most common being apoptosis, necrosis, and autophagy. Apoptosis is an endogenous mechanism involved with complicated apoptosis signaling cascade and can be compromised by augmented antiapoptotic signaling or loss of proapoptotic mechanisms [22]. Therefore, drug-resistance is induced in many chemotherapy-based cancer treatments. Ferroptosis, as an alternative approach of inducing cell death by lipid peroxidation, has attracted much attention to overcome some of the challenges associated to drug-resistant cancers [23]. Recently, ferroptosis has also been associated with PDT [16,24,25]. Our group has focused on the development of PSilQ nanoparticles as a photosensitizer-delivery platform for PDT [5,6,7,8,9,26]. We have already demonstrated that PSilQ nanoparticles carrying PpIX as a photosensitizer induce phototoxicity through apoptotic and necrotic cell death mechanisms [8]. However, the possibility of these nanoparticles also triggering ferroptosis has not been investigated. In this study, we synthesized PpIX-PSilQ nanoparticles without stimuli-responsive features to warrant permanent encapsulation of PpIX molecules in the nanoparticle. Based on an already established synthetic protocol in our group, we synthesized and characterized a PpIX silane derivative that is used for the fabrication of the PpIX-PSilQ nanoparticles in this work (Scheme S1) [7,9]. A three-component microemulsion method was used for the fabrication of the PpIX-PSilQ nanoparticles. The nanomaterial is spherical with a diameter of 41.7 ± 4.9 nm (Figure 1 and Figure S1), a hydrodynamic diameter of 262.6 ± 20.0 nm, and ζ-potential of −35.5 ± 4.0 mV (Table S1). The hydrodynamic diameter of the material is fairly constant over a period of 24 h, and minimal degradation was observed during 192 h as an indication of its colloidal and chemical stability (Figure S1B–C). As reported previously, PSilQ nanomaterials are distinguished for reaching a high loading capacity of the therapeutic agent [9,27,28,29]; in this case, the loading of PpIX to PSilQ nanoparticles was determined to be 24.0 ± 4.0%wt.

We evaluated the in-vitro properties of the PpIX-PSilQ nanoparticles in a human melanoma cell line, A375 cells. Several studies have showed promising results supporting the efficacy of PDT to treat melanoma either as primary or adjuvant therapy at different stages of the disease [30,31]. Flow cytometry and confocal microscopy showed that A375 cells were able to internalize the nanoparticles in high amounts despite the negative charge on their surface (Figure 1D–E). We previously reported that the cell internalization of PSilQ nanoparticles is usually carried out through an endocytosis pathway [8,9].

The PDT performance in A375 showed the typical dose response associated to the PpIX-PSilQ nanoparticles (Figure S3). In comparison with the parent porphyrin PpIX, the phototoxicity of the nanoparticles is reduced about nine times, IC_50_ for PpIX = 9.4 µM vs PpIX-PSilQ NPs = 81.2 µM. This difference can be explained by the self-quenching effect produced through encapsulating of photosensitizers in the nanoparticles, which directly impacts the generation of ^1^O_2_ [5,6]. Nevertheless, our group showed that by rendering stimuli-responsive degradability to the nanoparticles, the PDT capability can be restored, making this a promising approach to avoid unwanted side effects due to phototoxicity of photosensitizers in healthy tissue [8,9].

Cell death related to PDT is triggered by the production of reactive oxygen species inside the cells. First, we measured the generation of ROS by PpIX-PSilQ nanoparticles in vitro using DCFH-DA as ROS probe with flow cytometry. The quantification of positive cells associated with ROS demonstrated that nanoparticles in the presence of light generated ROS in a concentration-dependent manner. A similar trend was shown for PpIX (Figure 2A–B). Confocal microscopy was used to further demonstrate the ability of PpIX-PSilQ nanoparticles for ROS production (Figure S4A,B). DCFH-DA is a ROS probe specific for detecting hydrogen peroxide, hydroxyl, and peroxyl radicals in vitro [18,19]. The confocal micrographs corroborate the formation of ROS by PpIX-PSilQ NPs after activation with light.

Apoptosis is the most common cell death mechanism associated with PDT. The impact of light-activated photosensitizers on mitochondria is reported as a major factor controlling the induction of apoptosis. The caspase-dependent apoptotic cascade involving mitochondrial membrane depolarization and subsequent loss of cytochrome *c* (*cyt c*) has been well characterized with respect to PDT [32]. In cancer cells with abnormally high levels of *Bcl-2*, photodamage by cross-linking or cleavage may limit the effectiveness of this important anti-apoptotic control. In many cases, *Bcl2* has been reported as an important molecular target of PDT that promotes apoptosis in treated cells [33,34]. We previously reported enhancement in apoptosis resulting from silencing of *Bcl-2* gene in A375 cells [35]. In our previous work, we demonstrated that PpIX-based PSilQ nanoparticles use apoptosis as one of the main cell death mechanisms to eliminate triple-negative breast cancer cells [8]. Herein, we used the Annexin V apoptosis assay to confirm that apoptosis also plays an important role in the PDT of A375 cells using PpIX-PSilQ nanoparticles or the parent porphyrin PpIX (Figure 3A,B). We also observed that a higher proportion of necrotic cells were obtained for the PpIX-PSilQ nanoparticles. Nanoparticles, which are usually endocytosed and trafficked through the endolysosomal pathway, are highly localized in lysosomes [36]. It has been shown that the excess of ROS generated by PSs inside the lysosomal compartment prompt lysosomal membrane permeabilization (LMP) [37]. Excessive LMP often causes necrotic cell death by extreme cytosolic acidification [38]. Control experiments in the dark show a minimal number of apoptotic or necrotic cells associated with nanoparticles (Figure S5).

Ferroptosis is described as a regulated cell death mechanism driven by lipid peroxidation and the suppression of the GpX enzymatic activity, often observed as a consequence of excess intracellular ROS produced by Fenton reactions mediated by iron [13,14,15]. In this project, PDT affords lipid peroxidation by two different mechanisms: the direct reaction of ^1^O_2_ or the reaction of hydroxyl radicals with lipid membrane [16]. Both alternatives afford the accumulation of lipid hydroperoxides [39,40]. In addition, PDT can induce direct photo-oxidative inactivation of GpX enzymes by ^1^O_2_ and GSH depletion, which results in increased intracellular peroxides that are responsible for cellular damage through ferroptosis [17,41,42]. Therefore, PDT can trigger ferroptosis by increasing the amount of lipid peroxides and inactivating GpX enzymes. We investigated the effect of PpIX-PSilQ nanoparticles in both the formation of lipid peroxides and inactivation of GpX enzymes. The kinetic profile for the generation of NADP^+^, which is an indirect approach to measure the activity of GpX enzymes, clearly demonstrated a reduction in the production of NADP^+^ when A375 cells were treated with PpIX-PSilQ nanoparticles in the presence of light irradiation (Figure S6). A significant reduction of the reaction rate values for the generation of NADP^+^ confirms the impact of PpIX-PSilQ nanoparticles on the activity of GpX enzymes (Figure 3c). Similar performance was observed for PpIX molecules. The photo-oxidative mechanism for the inactivation of GpX enzymes by photosensitizers through type II reaction was reported using rose Bengal [41]. An irreversible oxidation of selenocysteine centers in GpX to dehydroalanine (DHA), and consequent loss of GpX activity due to ^1^O_2_ was detected in the lysates of J774A.1 cells photo-treated with this photosensitizer. Overproduction of ^1^O_2_ diminishes the activity of GpX enzymes most likely by modification of selenocysteine residues [43].

We also studied the formation of lipid peroxides using a lipid peroxidation sensor through flow cytometry and confocal microscopy. Similar to PpIX molecules, PpIX-PSilQ nanoparticles after light irradiation enhanced the generation of lipid peroxides compared with only light treatment (two-way ANOVA, *p* ≤ 0.001) (Figure 3D and Figure 4). Overall, our results show that by encapsulating PpIX molecules in the PSilQ platform, comparable trends were observed with PpIX on the inactivation of GpX enzymes and production of lipid peroxides as a clear indication that PpIX-PSilQ nanoparticles lead to ferroptosis as one of the mechanisms of cell death.

To further confirm that the ferroptosis mechanism is involved in the PDT performance of PpIX-PSilQ nanoparticles, we tested the phototoxicity of the material in the presence of ferrostatin-1, a selective and potent inhibitor of ferroptosis (Figure 5). This molecule is a radical-trapping antioxidant agent that traps peroxyl radicals in membrane lipids, which are the primary species to trigger ferroptosis [44]. The phototoxicity data showed that the presence of ferrostatin-1 reduced the PDT effect of PpIX-PSilQ nanoparticles and PpIX molecules by 36% and 63%, respectively. Our results described above show that the PDT effect of PpIX-PSilQ NPs reduced GpX activity, contributing to the failure of peroxyl radical-scavenging capacity and increasing the lipid peroxidation levels of treated cells. However, the addition of the phospholipid radical-trapping agent ferrostatin-1 reduced the phototoxicity exerted by PpIX-PSilQ NPs. This significant impact of ferrostatin-1 in the PDT performance of both PpIX-PSilQ nanoparticles and PpIX gives a clear indication that ferroptosis is an important cell death mechanism in their PDT performance [24,25,43,45]. Overall, these results are convincing arguments to support the hypothesis that ferroptosis is one of the main cell death mechanisms triggered by PSilQ nanoparticle-mediated photodynamic therapy.

## 5. Conclusions

We designed and fabricated PpIX-PSilQ nanoparticles with minimal leaking of the photosensitizer to study the cell death pathways associated with their PDT effect in A375 cells. PpIX-PSilQ nanoparticles followed similar cell death pathways as the parent PpIX photosensitizer. Apoptosis is an important pathway for PpIX and PpIX-PSilQ nanoparticles as well. PpIX-PSilQ nanoparticles showed higher necrotic cells than PpIX, most likely related to lysosomal membrane permeabilization, which is directly associated with the intracellular trafficking of the nanoparticles. We demonstrated, by analyzing the level of lipid peroxides and inactivation of GpX enzymes, that PpIX-PSilQ nanoparticles also follow ferroptosis as an important pathway to kill A375 cancer cells. This study provides a deeper understanding of the cell death pathways that account for the PDT effect of photosensitizer-loaded PSilQ nanoparticles. We envision that this investigation provides relevant results to develop promising light-activated nanoparticles that depend on ferroptosis for the treatment of apoptosis-resistant cancer cells.

## Data Availability

The data presented in this study are available within the article or supplementary materials.

## Supplementary Materials

The following are available online at https://www.mdpi.com/article/10.3390/nano11092324/s1: Materials and methods, Scheme S1: Synthesis of PpIX derivatives, Figure S1: SEM, DLS and chemical stability test, Figure S2: Phototoxicity and cytotoxicity of PpIX-PSilQ nanoparticles and PpIX in A375 cells, Figure S3: Intracellular ROS confocal images, Figure S4: Apoptotic and necrotic assay, Figure S5: GpX activity assay, Figure S6: Lipid peroxidation assay using confocal microscopy, Figure S7: Lipid peroxidation detected by BODIPY™ 581/591 C-11 sensor, Figure S8: Cytotoxicity of PpIX-PSilQ nanoparticles and PpIX in A375 cells in the absence or presence of Ferrostatin-1, and Table S1: Hydrodynamic diameter and PdI by DLS, ζ-potential and TGA data.
