# Supplementary material for: Light-Activated Protoporphyrin IX-Based Polysilsesquioxane Nanoparticles Induce Ferroptosis in Melanoma Cells
Source: Nanomaterials (Basel). 2021 Sep 7;11(9):2324. doi: 10.3390/nano11092324 (PMC8470003; doi:10.3390/nano11092324)
Supplement: Supplementary file 1 [file nanomaterials-11-02324-s001.zip › nanomaterials-1341896 SI Second Proof.pdf]

Light-Activated Protoporphyrin IX-Based Polysilsesquioxane Nanoparticles Induce Ferroptosis in Melanoma Cells

Hemapriyadarshini Vadarevu[1,2], Ridhima Juneja[1], Zachary Lyles[1,2], Juan L. Vivero-Escoto[1,2,3*]

[1]Department of Chemistry, The University of North Carolina at Charlotte, Charlotte, NC, U.S.A.; [2]Nanoscale Science Program, The University of North Carolina at Charlotte, Charlotte, NC, U.S.A.; [3]The Center for Biomedical Engineering and Science, The University of North Carolina at Charlotte, Charlotte, NC, U.S.A.

Correspondence: Juan Luis Vivero-Escoto

Department of Chemistry, The University of North Carolina at Charlotte, Charlotte NC 28223, U.S.A.

Tel: +1 704 687 5239

Fax: +1 704 687 0960

Email: Juan.Vivero-Escoto@uncc.edu

Electronic Supporting information

Materials

Protoporphyrin IX (PpIX), dioctyl sodium sulfosuccinate (Aerosol OT or AOT), 2',7'-dichlorodihydrofluorescein diacetate (DCFH-DA), BODIPY™ 581/591 C-11 (Lipid peroxidation sensor) and SYTOX™ Blue dead cell stain and Glutamax were obtained from Thermo Fischer Scientific (Waltham, Massachusetts, USA). 1- ethyl-3-(3-dimethylaminopropyl) carbodiimide (EDC) was purchased from Oakwood Chemical (Columbia, South Carolina, USA). N-butanol, dithiothreitol (DTT), serine, 3-(Triethoxysilyl)propyl isocyanate (TESPIC), Ferrostatin-1, sterile-filtered DMSO and the rest of the chemicals used in this work were obtained from Sigma-Aldrich

(St. Louis, Missouri, USA). Chemicals and solvents were used without any further purification unless specified otherwise. Roswell Park Memorial Institute (RPMI 1640), Dulbecco Modified Eagle Medium (DMEM), penicillin-streptomycin (pen-strep), phosphate buffer saline (PBS, 1X), and trypsin were purchased from Corning (Corning, New York, USA). CellTiter 96® AQueous Assay was obtained from Promega (Madison, WI, USA). Non-essential amino acids (NEAA) was purchased from Quality biologicals (Gaithersburg, Maryland, USA). Fetal bovine serum (FBS) was purchased from Atlanta Biologicals (Atlanta, Georgia, USA). Hoechst 33342 dye was purchased from Life Technologies (Waltham, Massachusetts, USA). BD Pharmingen™ Annexin V-FITC apoptosis detection kit was purchased from BD Biosciences (San Jose, California, USA). Glutathione peroxidase activity assay kit (Fluorometric) was purchased from Abcam (Waltham, Massachusetts, USA).

Methods

A Raith 150 Field Emission Scanning Electron Microscope (SEM) (Raith America Inc., New York, NY, USA) was utilized to measure the particle size and shape of the materials. Nanoparticle samples were suspended in methanol in preparation for the SEM. Dynamic light scattering (DLS) and ζ-potential measurements were carried out using a Malvern Instrument Zetasizer Nano (red laser 633 nm) (Malvern Instrument Ltd., Malvern, UK). The amount of PpIX loaded into the PSilQ NPs was quantified by thermogravimetric analysis (Mettler-Toledo AG Analytical, Schwersenbach, Switzerland). The thermal degradation profiles were obtained for a heating rate of 1 °C/min between 25 and 800 °C followed by a 60 min hold at 800 °C. Biotable power source with RGB LED array (MM Optics, University of São Paulo, Brazil) emitting at 630 nm (24.5 mW/cm^2) was used for all *in vitro* PDT experiments. A microplate reader (TECAN Spark) was used for fluorescence intensity measurement in kinetic mode for $NADP^+$ (San Jose, California, USA). A Multiskan FC plate reader by Fisher Scientific plate reader was used for the cell viability

analysis (Waltham, Massachusetts, USA). A BD LSRFortessa™ cell analyzer was used for the fluorescence-activated cell sorting (FACS) experiments (Indianapolis, Indiana, USA). An Olympus Fluoview FV 1000 confocal laser scanning microscope (CLSM) was used for the confocal experiments (Center Valley, Pennsylvania, USA).

Synthesis of PpIX silane derivative (4)

The synthesis of the PpIX silane derivative used as monomer for the fabrication of PpIX-PSilQ nanoparticles was performed in a three-step reaction pathway (Scheme S1). First, the synthesis of protoporphyrin IX succinimide ester (PpIX-SE) (**2**) was carried out by placing 0.889 mmol (500 mg) of protoporphyrin IX (PpIX) (**1**) in a round bottom flask containing 20 mL of dimethyl sulfoxide (DMSO) and 70 mL of dichloromethane (DCM). To this solution, 2.226 mmol (272 mg) of 4-dimethyl amino pyridine was added along with a solution of 5.320 mmol (1.02 g) of 1-ethyl-3-(3-dimethylaminopropyl) carbodiimide (EDC) hydrochloride in 20 mL of DMSO. The flask was then placed in an ice bath and stirred for 10 min. To this mixture, 5.326 mmol (613 mg) of N-hydroxysuccinimide (NHS) was added to the flask, and was kept in an ice bath for 3 h. A diluted ethanolic solution in water (100 mL, EtOH:H_2O/75:25% vol./vol.) was added to the flask to afford precipitation of the product. PpIX-SE (**2**) was then collected through gravity filtration and washed with ethanol before being dried in a lyophilizer. Yield: 506 mg (76 %wt.); ^{1}H NMR (DMSO-d_6, ppm): δ 2.71 (m, 8H), 3.15 (t, 4H, J = 7.3 Hz), 3.55 (s, 3H), 3.56 (s, 3H), 3.61 (s, 3H), 3.63 (s, 3H), 4.28 (t, 4H, J = 7.3 Hz), 6.17 (d, 2H, J = 11.7 Hz), 6.38 (d, 2H, J = 17.9 Hz), 8.36 dd, 1H, J = 17.2 Hz, 11.7 Hz), 8.44 (dd, 1H, J = 17.2 Hz, 11.7 Hz), 10.05 (s, 1H), 10.09 (s, 1H), 10.10 (s, 1H), 10.12 (s, 1H). FT-IR (cm^{-1}): 3503 (N-H), 2915 (C-H), 1808 (C=O), 1778 (C=O), 1732 (C=O), 1627 (CN); MALDI-MS (m/z): Calculated: $[M]^+$ = 756.30; Observed: $[M]^+$ = 756.31. UV-Vis (DMF, nm): 404 (S-band); 623, 576, 542, 506 (Q-bands). Molar extinction coefficient (λ = 404 nm; DMF; mol L^{-1} cm^{-1}): 195,800. As the second step, PpIX-serine (**3**) was afforded by dissolving 0.46 mmol (350 mg)

of PpIX-SE (2) in 25 mL of DMSO. To this mixture, an aqueous solution of serine (1.5 mmol, 150 mg in 3.0 mL of water) was added by the addition of diisopropylethylamine (DIPEA) (2.0 mmol, 350 μL). The final solution was heated and stirred at 120 °C for 72 h. A diluted acidic ethanolic-based solution (250 mL of H_2O:Ethanol; 20:80 %vol./vol.; 200 μL of concentrated HCl) was added to the flask to precipitate out the product. The precipitate is collected by gravity filtration and washed with water before being dried in a lyophilizer. Yield: 506 mg (49 %wt.); ^1H NMR (DMSO-d_6, ppm): 3.06 (t, 4H, J = 7.7 Hz), 3.54 (s, 3H), 3.56 (s, 3H), 3.62 (s, 3H), 3.64 (s, 3H), 3.92 (d, 4H, J = 5.1 Hz), 4.34 (t, 4H, J = 7.7 Hz), 4.66 (t, 2H, J = 5.1 Hz), 6.14 (d, 2H, J = 11.1 Hz), 6.33 (d, 2H, J = 17.4 Hz), 8.39 (dd, 1H, J = 17.4 Hz, 11.1 Hz), 8.47 (dd, 1H, J = 17.4 Hz, 11.1 Hz), 10.04 (s, 1H), 10.12 (s, 1H), 10.18 (s, 1H), 10.21 (s, 1H). IR (cm^{-1}): 3306 (N-H), 2920 (C-H), 1710 (C=O), 1640 (C-N). UV-Vis (DMF, nm): 408 (S-band); 628, 575, 540, 506 (Q-bands). Molar extinction coefficient (λ = 408 nm; DMF; mol L^{-1} cm^{-1}): 75,800. To produce the PpIX silane precursor (**4**), PpIX-serine (**3**) (0.13 mmol, 100 mg) was placed into a round bottom flask under N_2 atmosphere followed by the addition of dry DMF (10 mL). To this solution, triethylamine (0.57 mmol, 161.8 μL) was slowly added until compound (**3**) was fully dissolved. Later, the whole solution was placed in an ice bath followed by the addition of triethoxysilyl propyl isocyanate (TESPIC) (0.27 mmol, 134.7 μL). The final solution was left for 3 h in the ice bath, let warmed up to room temperature and stirred for an additional 15 h. To obtain the final product, 80 mL of acidic aqueous solution (200 μL of conc. HCl) was added to precipitate out compound **4** and collected it by gravity filtration. The PpIX silane derivative is dried in a lyophilizer. ^1H NMR (DMSO-d_6, ppm): 0.24 (t, 4H, J = 8.1 Hz), 0.90 (t, 18H, J = 6.9 Hz), 1.25 (q, 4H, J = 8.1 Hz), 3.00 (t, 4H, J = 7.7 Hz), 3.06 (t, 4H, J = 8.1 Hz), 3.43 (q, 12H, J = 6.9 Hz), 3.56 (s, 3H), 3.58 (s, 3H), 3.67 (s, 3H), 3.70 (s, 3H), 3.92 (d, 4H, J = 5.1 Hz), 4.34 (t, 4H, J = 7.7 Hz), 4.66 (t, 2H, J = 5.1 Hz), 6.12 (d, 2H, J = 11.3 Hz), 6.31 (d, 2H, J = 17.9 Hz), 8.36 (dd, 1H, J = 17.9 Hz, 11.3 Hz), 8.44 (dd, 1H, J = 17.9 Hz, 11.3 Hz), 10.04 (s, 1H), 10.12 (s, 1H), 10.18

(s, 2H). IR (cm^{-1}): 3302 (N-H), 2923 (C-H), 1709 (C=O), 1648 (C-N), 1066-1102 (Si-O; Si-C). UV-Vis (DMF, nm): 410 (S-band); 629, 576, 541, 507 (Q-bands). Molar extinction coefficient (λ = 410 nm; DMF; mol L^{-1} cm^{-1}): 55,400.

Quantification of the amount of PpIX loaded to PpIX-PSilQ nanoparticles using UV-vis spectroscopy

UV-Visible spectroscopy was used to confirm the amount of PpIX loaded to the PpIX-PSilQ NPs. Spectral absorbance of PpIX-PSilQ NPs dispersed in 3 mL of DMSO was measured at λ_{max} = 401 nm using Varian Cary Bio50 UV-Vis absorption spectrophotometer. The obtained absorbance value was fitted using PpIX calibration curve in the same solvent (Figure S2). The loading percentage was calculated using the fitting equation, the volume of DMSO and the original mass of nanoparticles in the sample.

Leakage of PpIX from PpIX-PSilQ NPs

PpIX-PSilQ NPs was dispersed in DMF at a concentration of 2 mg/mL to determine the amount of PpIX leaked. The dispersion was stirred at 37 °C in the presence or absence of 10 mM DTT. The release of PpIX from the nanoparticles was measured by recording the absorbance of supernatants collected at 408 nm. The supernatant was returned to the original vial, and the nanoparticles were re-dispersed after each measurement. The measurements were taken at staggered intervals of time for a period of 8 days. The absorbance values were used to calculate the amount of PpIX leaked from the PpIX-PSilQ nanoparticles using a calibration curve of PpIX in DMF.

***In Vitro* Phototoxicity of PpIX-PSilQ nanoparticles**

The viability of A375 cells after PDT treatment with PpIX-PSilQ NPs or PpIX was analyzed by the MTS assay. The cells were seeded in 96 well plates at a density of 2,000 cells/well in 100 μL of complete DMEM and incubated at 37 °C in 5% CO_2 atmosphere for 24 h. After media removal,

PpIX (0.1-100 μM) or PpIX-PSilQ NPs (at equivalent PpIX concentrations of 10-200 μM) were added to the cells in fresh media. After 48 h of incubation, cells were washed once with DPBS and irradiated with red light (630 nm, 24.5 mW/cm^2) for 20 min. Treated cells were replenished with 100 μL of fresh media and incubated for additional 24 h. Control dark experiments were conducted in parallel with PpIX-PSilQ NPs or PpIX at the same concentrations but were maintained in the dark for the entire duration of the experiment. To determine the phototoxicity of the PDT treatment, the cells were washed once with DPBS and 100 μL media was added along with 20 μL of CellTiter 96 solution to each well. The cells were incubated for 2 h at 37 °C in 5% CO_2 atmosphere. Cell viability (%) was calculated by analyzing absorbance values recorded at 490 nm using a microplate reader. Cell viability (%) was calculated as follows: viability = $(A_{sample} - A_{blank})/(A_{control} - A_{blank}) \times 100\%$, where A_{sample} , $A_{control}$ and A_{blank} denote absorbance values of the sample, control, and blank wells. The IC_{50} values are determined using GraphPad Prism (v8.3.0 for Windows, La Jolla, CA, USA), fitting the normalized viability data to a nonlinear regression.

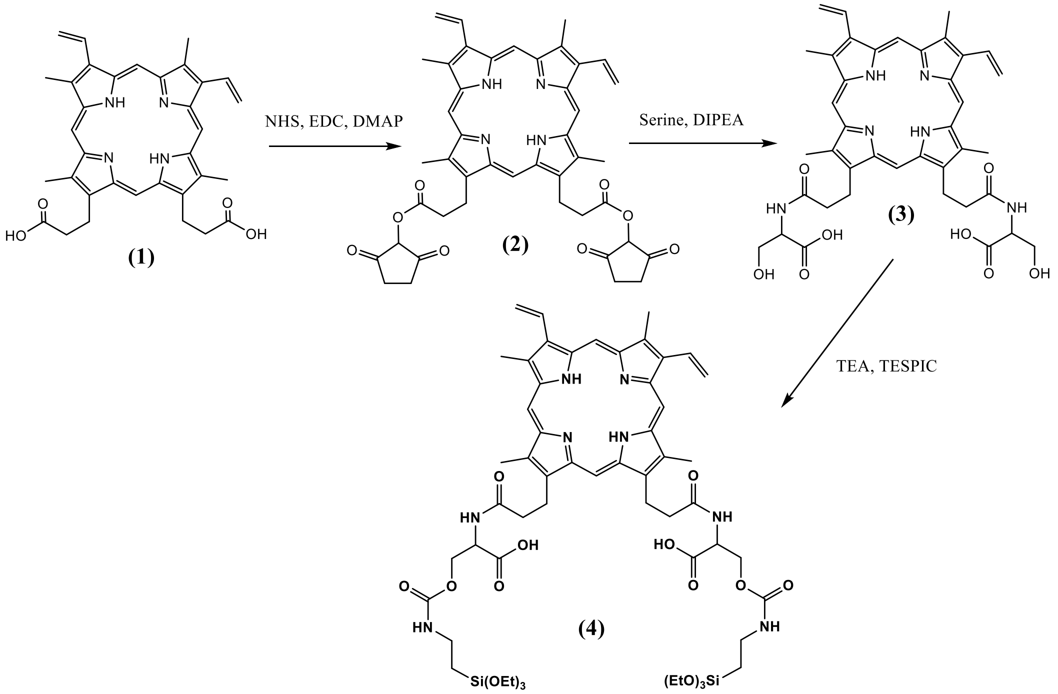

Scheme S1. The carboxylic acid groups were activated through an EDC/NHS coupling reaction to afford compound **2**. Following activation, molecule **3** was synthesized by adding serine as a trifunctional linker. Finally, the isocyanate silane precursor reacted with the alcohol group through a nucleophilic acyl reaction to produce the PpIX silane monomer **4** used for the fabrication of PpIX-PSilQ NPs.

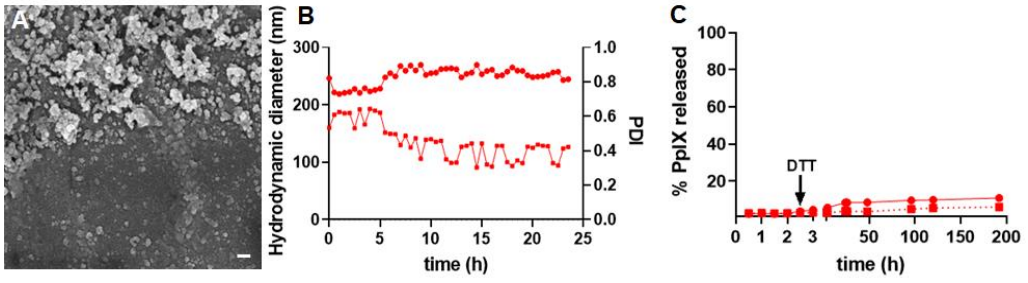

Figure S1. (**A**) SEM image of PpIX-PSilQ NPs. Scale bar= 200 nm. (**B**) Colloidal stability of the PpIX-PSilQ NPs in complete cell culture media supplemented with serum for 24 h monitored using DLS, hydrodynamic sizes are represented by (circles) and PdI represented as (squares). (**C**) Leakage of PpIX in the presence (circles) and absence (squares) of reducing agent (DTT).

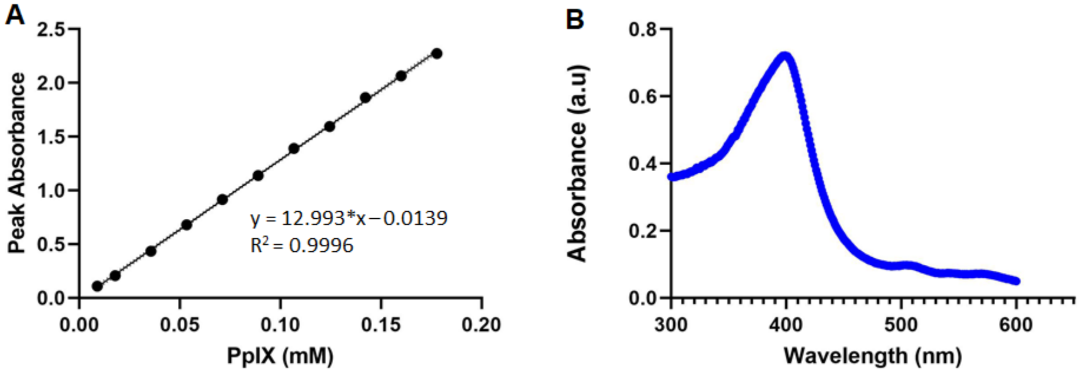

Figure S2. (**A**) Calibration curve of PpIX in DMSO (λ_{max} = 401 nm). (**B**) UV-vis spectrum of PpIX-PSilQ nanoparticles in DMSO.

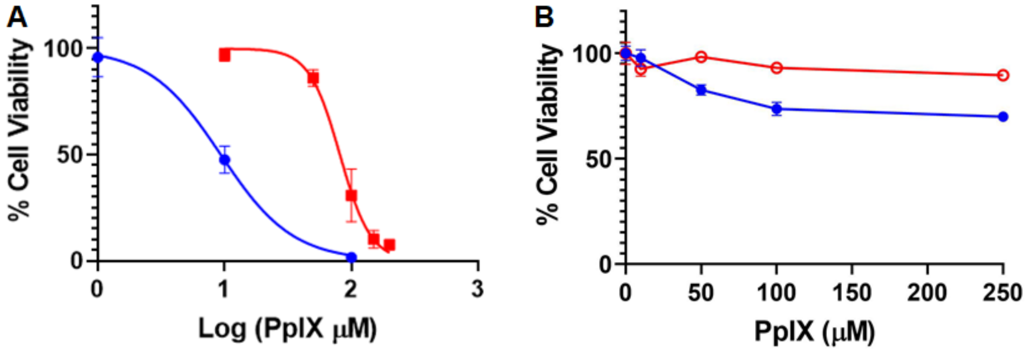

Figure S3. (**A**) Phototoxicity and (**B**) cytotoxicity of PpIX-PSilQ nanoparticles (red) and PpIX (blue) in A375 cells. Irradiation with red light (630 nm; 24.5 mW cm^{-2}) for 20 min (n = 6).

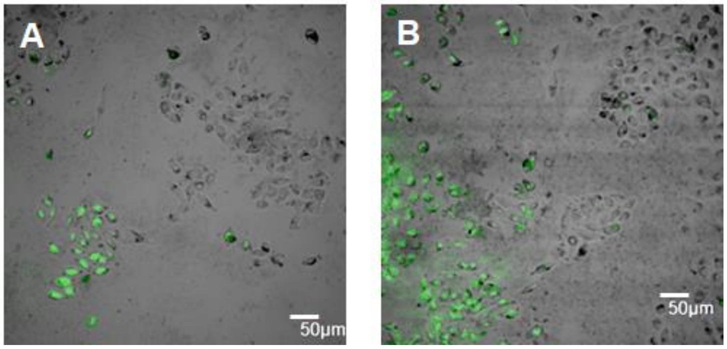

Figure S4. Intracellular ROS production in A375 cells using confocal microscopy for **(A)** PpIX-PSilQ nanoparticles (25 μM equivalent of PpIX) and **(B)** PpIX (25 μM). Images are an overlap of the green channel (DCF fluorescence) and brightfield. Scale bar = 50 μm.

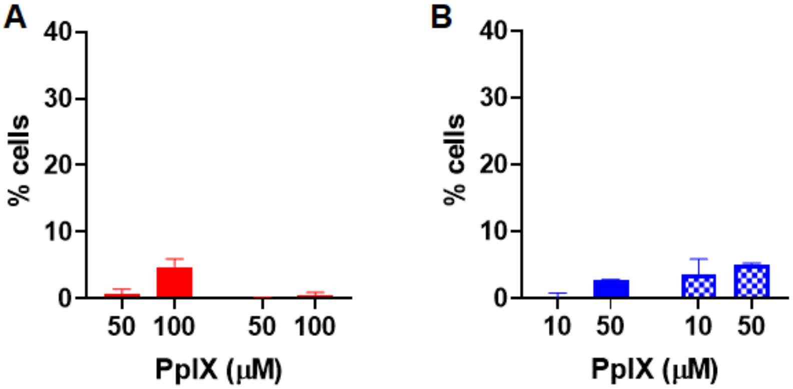

Figure S5. (**A**) Apoptotic (red solid bars) and necrotic (red checkered bars) cells treated with PpIX-PSilQ NPs (50 and 100 μM equivalent of PpIX) in the absence of light. (**B**) Apoptotic (blue solid bars) and necrotic (blue checkered bars) cells treated with PpIX (10 and 50 μM) in the absence of light. Data are represented as mean ± SD.

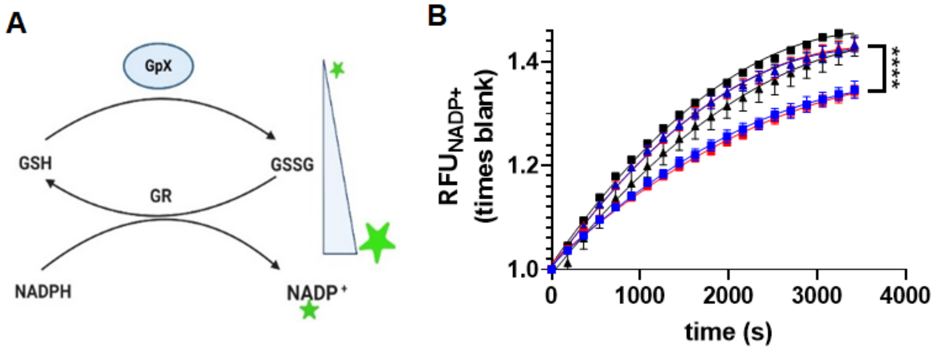

Figure S6. Inactivation of glutathione peroxidase and lipid peroxidation by PpIX-PSilQ nanoparticles. (**A**) Schematic depiction of the working principle of the GpX activity assay. The generation of $NADP^+$ is used to indirectly determine the catalytic activity of GpX. (**B**) Kinetic profiles of $NADP^+$ production for irradiated samples: control (black squares), PpIX-PSilQ NPs (50 µM PpIX eq.) (red squares), PpIX (50 µM) (blue squares); and non-irradiated samples: control (black triangles), PpIX-PSilQ NPs (50 µM PpIX eq.) (red triangles), and PpIX (50 µM) (blue triangles). Data are represented as mean ± SD. Statistics: Two-way ANOVA using Tukey's multiple comparison test: ****$p \leq 0.0001$, ***$p \leq 0.001$, **$p \leq 0.01$, *$p \leq 0.05$, and ns: $p > 0.05$.

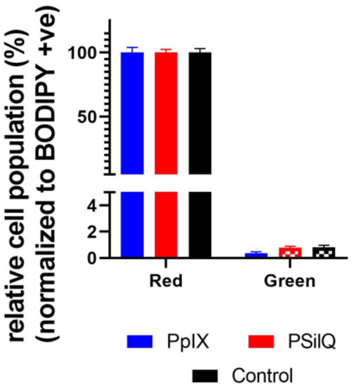

Figure S7. Quantification of lipid peroxidation using confocal microscopy in A375 cells treated with control (black), PpIX-PSilQ NPs (50 µM PpIX eq.) (red), and PpIX (50 µM) (blue) in the red (solid bars) and green (dashed bars) channels. Data are represented as mean ± SD.

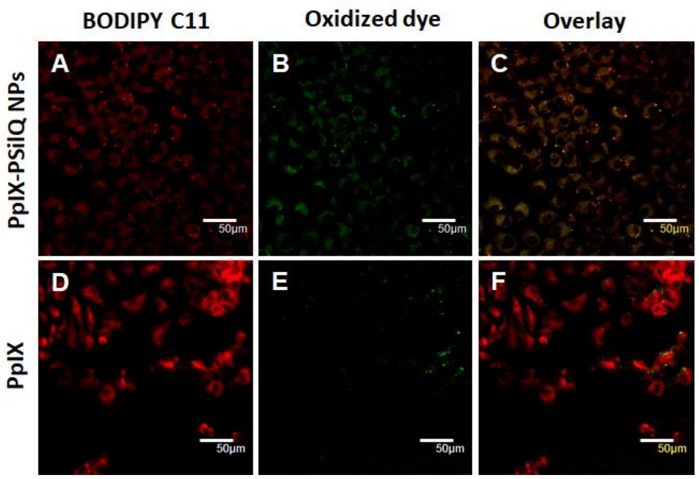

Figure S8. Lipid peroxidation detected by BODIPY™ 581/591 C-11 sensor. Confocal micrographs of A375 cells inoculated with (**A**-**C**) PpIX-PSilQ nanoparticles or (**D**-**F**) PpIX in the absence of light. The red channel depicts the presence of BODIPY 581/591 C-11 (**A**/**D**), the green channel shows the fluorescence corresponding to the oxidized version of BODIPY 581/591 C-11 (**B**/**E**), merged image of the red and green channels (**C**/**F**).

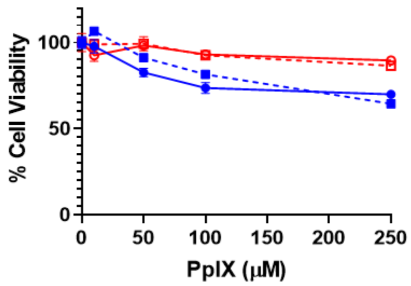

Figure S9. Cytotoxicity of PpIX-PSilQ nanoparticles (red) and PpIX (blue) in A375 cells in the absence (solid) or presence (dashed) of Ferrostatin-1 (2 µM) (n = 6).

Table S1. Hydrodynamic diameter and PdI by DLS, ζ-potential and TGA data.

DLS		
	Size/nm (PDI)	ζ-potential (mV)
PpIX-PSilQ NPs	262.6 ± 20.0 (0.40)	-35.5 ± 4.0

Material	Aromatics (%)
PpIX	62.5
PpIX Ligand 4	63.0
PpIX-PSilQ NPs	24.0 ± 2.0
